# PD-L1 Protein Expression in Middle Eastern Breast Cancer Predicts Favorable Outcome in Triple-Negative Breast Cancer

**DOI:** 10.3390/cells10020229

**Published:** 2021-01-25

**Authors:** Sandeep Kumar Parvathareddy, Abdul K. Siraj, Saeeda O. Ahmed, Laila Omar Ghazwani, Saud M. Aldughaither, Fouad Al-Dayel, Asma Tulbah, Dahish Ajarim, Khawla S. Al-Kuraya

**Affiliations:** 1 Human Cancer Genomic Research, King Faisal Specialist Hospital and Research Center, P.O. Box 3354, Riyadh 11211, Saudi Arabia; psandeepkumar@kfshrc.edu.sa (S.K.P.); asiraj@kfshrc.edu.sa (A.K.S.); ahmsaeeda@kfshrc.edu.sa (S.O.A.); laila.omar202020@hotmail.com (L.O.G.); saud_d@hotmail.com (S.M.A.); 2 Department of Pathology, King Faisal Specialist Hospital and Research Centre, P.O. Box 3354, Riyadh 11211, Saudi Arabia; dayelf@kfshrc.edu.sa (F.A.-D.); tulbah@kfshrc.edu.sa (A.T.); 3 Department of Oncology Centre, King Faisal Specialist Hospital and Research Centre, P.O. Box 3354, Riyadh 11211, Saudi Arabia; ajarim@kfshrc.edu.sa

**Keywords:** PD-L1, breast cancer, triple negative breast cancer, prognosis

## Abstract

Programmed cell-death ligand 1 (PD-L1) has been shown to induce potent T-cell mediated anti-tumoral immunity. The significance of PD-L1 expression in the prognosis of breast cancer (BC) remains controversial and its prevalence and prognostic value in breast cancer from Middle Eastern ethnicity is lacking. A total of 1003 unselected Middle Eastern breast cancers were analyzed for PD-L1 expression using immunohistochemistry. PD-L1 expression, seen in 32.8% (329/1003) of cases, was significantly associated with poor prognostic indicators such as younger patients, high-grade tumors, estrogen-receptor (ER)-negative, progesterone-receptor (PR)-negative, and triple-negative breast cancers (TNBC) as well as high Ki-67 index. We also found a significant association between PD-L1 expression and deficient mismatch repair protein expression. No association was found between PD-L1 expression and clinical outcome. However, on further subgroup analysis, PD-L1 expression was found to be an independent marker for favorable overall survival and recurrence-free survival in TNBC. In conclusion, we demonstrated strong association between PD-L1 and mismatch repair deficiency in Middle Eastern BC patients and that PD-L1 overexpression in tumor cells was an independent prognostic marker in TNBCs from Middle Eastern ethnicity. Overall, these findings might help in the development of more appropriate treatment strategies for BC in Middle Eastern population.

## 1. Introduction

Breast cancer (BC) incidence in Saudi Arabia is on the rise. A unique characteristic of breast cancer in this population is the relatively younger age of disease onset, where a large number of patients present with invasive ductal carcinoma before the age of 50 years [1]. Despite the advances in therapeutic modalities for BC, distant metastasis or recurrence have occurred in more than 50% of patients with invasive breast cancer, resulting in treatment failure [2,3]. Therefore, there is an urgent need to identify molecular biomarker targets that could help in introducing new therapeutic approaches to specific patient sub-groups.

Immunotherapy is one of the most encouraging finding of cancer therapy in recent years [4,5,6]. One of the most common mechanisms underlying immunotherapy is programmed cell-death protein-1 (PD-1) and programmed cell-death ligand-1 (PD-L1), which serve as immune checkpoints in the tumor microenvironment [7,8]. Upon activation, the PD-1/PD-L1 axis induces functional impairment of antigen-specific T-cells, thus shielding the tumor cells from T-cell mediated killing [9,10,11]. PD-1/PD-L1 pathway inhibitors have achieved great success in clinical trials for patients with various types of cancer, and have been approved for use in clinical practice for patients with several cancers, including triple-negative breast cancer (TNBC) [7,12,13,14,15].

PD-L1 expression has been reported as an important prognostic biomarker in multiple studies, although its prognostic significance varied according to tumor type [16,17,18,19]. In breast cancer, association of PD-L1 overexpression with prognosis has revealed conflicting data. While some reports have noted that overexpression of PD-L1 is associated with worse prognosis [20,21,22], others have found PD-L1 expression to be associated with favorable prognosis and correlated with longer disease-free survival, especially in TNBC patients [23,24,25,26]. Furthermore, the prevalence and the predictive role of PD-L1 expression in breast cancer from Middle Eastern ethnicity has not been explored previously.

Therefore, we conducted this study to evaluate PD-L1 expression on more than 1000 breast cancer tissues from Middle Eastern ethnicity, surgically removed in a single institute, and assessed the correlation of PD-L1 expression with several clinico-pathological and molecular markers. Furthermore, the effect of PD-L1 on clinical outcome was explored to determine its potential as a biomarker for Middle Eastern BC patients’ prognosis.

## 2. Materials and Methods

### 2.1. Patient Samples and Data Collection

One thousand and nine patients with breast cancer diagnosed between 1990 and 2011 were selected from the files of the King Faisal Specialist Hospital and Research Centre (KFSHRC). The patients included in this study had their diagnosis, treatment, and follow-up care in the Department of Surgical Oncology at KFSHRC. The histologic subtype of each breast tumor sample was determined according to World Health Organization (WHO) criteria. Detailed clinico-pathological data, including follow-up data, were noted from case records and summarized in Table 1. Waiver of consent was obtained for the study from the Institutional Review Board and Research Ethics Committee of KFSHRC under Project RAC# 2140 008 on breast cancer archival clinical samples.

### 2.2. Tissue Microarray (TMA) Construction

TMA construction was performed as described earlier [27]. Briefly, tissue cylinders with a diameter of 0.6 mm were punched from representative tumor regions of each donor tissue block and brought into a recipient paraffin block using a modified semiautomatic robotic precision instrument (Beecher Instruments, Woodland, WI). Two cores of breast cancer were arrayed from each case.

### 2.3. Immunohistochemistry (IHC) Staining and Evaluation

Standard protocol was followed for manual IHC staining. For antigen retrieval, Dako (Dako Denmark A/S, Glostrup, Denmark) Target Retrieval Solution pH 9.0 (Catalog number S2368) was used, and the slides were placed in a Pascal pressure cooker at 120 °C for 10 min. Primary antibody against PD-L1 (E1L3N, 1:50 dilution, pH 9.0, Cell Signaling Technology, Danvers, MA) was used. The Dako Envision Plus System kit was used as the secondary detection system with 3, 30-diaminobenzidine as chromogen. All slides were counterstained with hematoxylin, dehydrated, cleared, and mounted. Normal tissues of different organ systems were also included in the TMA to serve as positive controls. Negative control was performed by omission of the primary antibody. Only freshly cut slides were stained simultaneously to minimize the influence of slide aging and maximize reproducibility of the experiment. The slides were independently examined by two pathologists. If there was a discrepancy in the individual scores, both pathologists carried out a re-evaluation until a consensus was reached.

A membranous and/or cytoplasmic staining was observed. Only the membrane staining was considered for scoring. PD-L1 was scored as described previously [28]. Scoring was done for tumor cells only. Briefly, the proportion of positively stained cells were calculated as a percentage for each core and the scores were averaged across two tissue cores from the same tumor to yield a single percent staining score representing each cancer patient. For the purpose of statistical analysis, the scores were dichotomized. Cases showing expression level of ≥5% were classified as positive for PD-L1 and those with less than 5% as negative.

Staining and scoring of estrogen-receptor (ER), progesterone-receptor (PR), Her-2 neu, Ki-67, and mismatch repair proteins was performed as described previously [29,30,31].

### 2.4. Statistical Analysis

The associations between clinico-pathological variables and protein expression was performed using contingency table analysis and Chi-square tests. The Mantel–Cox log-rank test was used to evaluate overall survival and recurrence-free survival. Survival curves were generated using the Kaplan–Meier method. The Cox proportional hazards regression model was used for multivariate analysis. Age, histologic subtype, tumor grade, lymph node metastasis, and tumor stage were included as covariates, since they are well-known prognostic factors in breast cancer. Two-sided tests were used for statistical analyses with a limit of significance defined as *p* value < 0.05. Data analyses were performed using the JMP11.0 (SAS Institute, Inc., Cary, NC) software package. 

## 3. Results

### 3.1. PD-L1 Expression in Breast Cancer and Its Clinico-Pathological Associations

**Figure 1:**
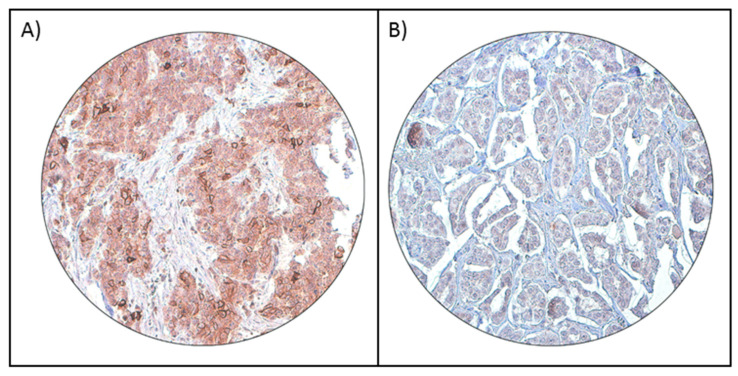
Programmed cell-death ligand 1 (PD-L1) immunohistochemical staining in breast cancer tissue microarray (TMA). Representative examples of tumors showing (**A**) positive and (**B**) negative (right panel) membrane staining of PD-L1 (20 X/0.70 objective on an Olympus BX 51 microscope (Olympus America Inc, Center Valley, PA, USA)).

**Figure 2:**
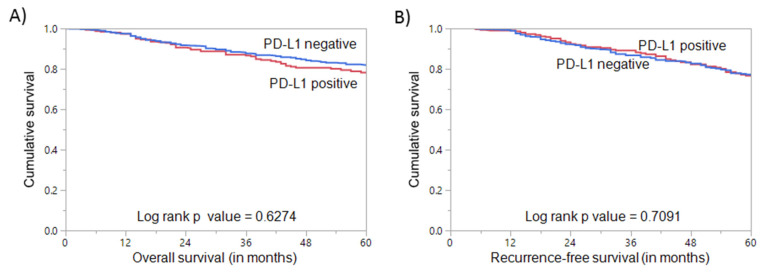
Survival analysis of PD-L1 protein expression in breast cancer. Kaplan–Meier survival plot showing no statistically significant difference between PD-L1 positive and negative tumors for (**A**) overall survival (*p* = 0.6274) and (**B**) recurrence-free survival (*p* = 0.7091).

PD-L1 protein expression was analyzed immunohistochemically in 1009 BC samples. However, six cases were excluded due to missing tissue cores in the TMA. Hence, 1003 samples were included for further analysis. PD-L1 expression ranged from 0–100% (median = 0%). Using a cut-off of ≥5%, PD-L1 expression was noted in 32.8% (329/1009) of BC (Figure 1A,B) and found to be associated with adverse clinico-pathological parameters such as younger age (*p* = 0.0432), higher grade (*p* = 0.0025), ER-negative (*p* < 0.0001), PR-negative (*p* = 0.0001), and triple-negative (*p* = 0.0062) breast cancers, as well as a high proliferative index (Ki-67) (*p* < 0.0001). We also found a significant association between PD-L1 expression and deficient mismatch repair (dMMR) protein expression (*p* = 0.0009) (Table 2). However, no significant association was found between PD-L1 expression and overall survival (OS) (*p* = 0.6274) or recurrence-free survival (RFS) (0.7091) in the entire cohort (Figure 2).

### 3.2. PD-L1 Expression in Triple-Negative Breast Cancer

Since several previous studies have noted an association between PD-L1 expression and TNBC, we sought to analyze the clinico-pathological associations and prognostic impact of PD-L1 in this sub-group of BCs. PD-L1 over-expression was seen in 45.0% (67/149) of TNBCs and was significantly associated with lymph node metastasis (*p* = 0.0459) (Table 3). No associations were found with other clinico-pathological variables.

### 3.3. PD-L1 Expression and Clinical Outcome in Triple Negative Breast Cancer

**Figure 3:**
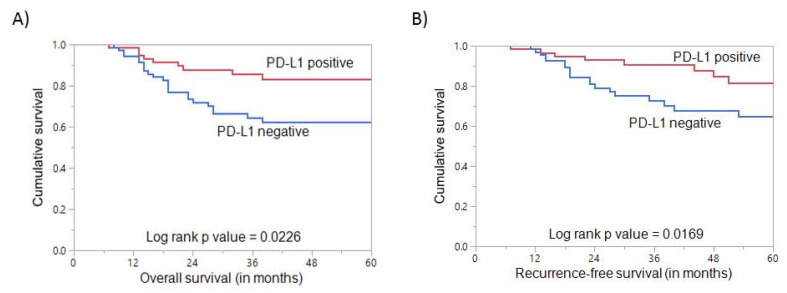
Survival analysis of PD-L1 protein expression in triple negative breast cancer. (**A**) Kaplan–Meier survival plot showing statistically significant good overall survival in PD-L1 positive tumors compared to PD-L1 negative (*p* = 0.0226) (**B**) Kaplan–Meier survival plot showing statistically significant good recurrence-free survival for PD-L1 positive tumors (*p* = 0.0169).

PD-L1 positive TNBCs were found to have a favorable impact on OS (*p* = 0.0226, Table 3, Figure 3A). On multivariate analysis, PD-L1 was an independent prognostic indicator of OS (HR = 0.28, 95% CI = 0.11–0.64, *p* = 0.0043) (Table 4). Patients with PD-L1 positive TNBCs were also found to have a favorable RFS (*p* = 0.0169, Table 3, Figure 3B). On multivariate analysis, PD-L1 expression was also an independent predictor of favorable RFS (HR = 0.31, 95% CI = 0.13–0.67, *p* = 0.0043) (Table 4).

## 4. Discussion

It has become widely recognized that the interaction of PD-1 and PD-L1 plays an important role in immune evasion by tumors, and PD-L1 expression in tumor tissue may be a good marker to predict the efficacy of anti-PD-L1 antibodies [32,33]. The importance of PD-L1 expression in tumor tissues as prognostic marker has not reached consensus, and data on its prognostic value in BC from Middle Eastern ethnicity is completely lacking.

In this study, we analyzed PD-L1 expression and its association with clinico-pathological characteristics. PD-L1 expression was seen in 32.8% (329/1003) of the BC cases, which is in alignment with reported PD-L1 positivity rate ranging from 17%–56% in existing literature [23,34,35,36,37]. Two of these five studies reported PD-L1 expression in whole tissue sections, whereas the other three studies analyzed PD-L1 expression in TMAs. This variation reported in PD-L1 expression can be attributed to sample size, variations in tissue preparation, use of different antibody clones, cut-off values and interpretation of IHC results. By analyzing clinico-pathological data, we found significant correlation between PD-L1 expression and higher grade (*p* = 0.0025), younger patients (*p* = 0.0432), higher Ki-67 index (>30%, *p* < 0.0001), hormone-receptor-negative (ER–PR) tumors (*p* = 0.0001), and TNBC (*p* = 0.0062). Despite the association with poor prognostic features observed in the overall cohort, we were unable to establish an association between tumor PD-L1 positivity and clinical outcome (OS and RFS). Importantly, PD-L1 positivity was significantly correlated with dMMR. Analogously, significant association between PD-L1 protein expression and dMMR was observed in a previous report [38]. This association is of important clinical relevance since dMMR seems to have strong connection with PD-L1 expression and PD-1/PD-L1 blockade therapy as evidenced by the fact that pembrolizumab was approved for many types of solid tumors with dMMR [39,40].

Upon further stratification, based on breast cancer subtype, PD-L1 expression as expected was higher in the TNBC than in other subtypes. This can be explained by the increased immunogenicity of TNBC which has been previously reported [41]. Most importantly, PD-L1 expression in TNBC subgroup was inversely associated with lymph node metastasis and significantly associated with favorable overall survival and better disease-free survival. This association with favorable patient outcome remains significant when considering all the factors using multivariate analysis. One explanation for the favorable prognosis could be due to a lower proportion of M1 patients and a higher fraction of stage I patients in the PD-L1 positive group. Recent studies have reported that PD-1, but not PD-L1, predicted good prognosis in TNBCs [42,43]. Additional studies also reported positive prognostic and predictive values of PD-L1 expression in immune and/or tumor cells in TNBC [25,26,44]. On the contrary, a study from China [45], which also analyzed PD-L1 expression in tumor cells alone, showed a worse prognosis in TNBC patients expressing PD-L1. In our study, we focused on PD-L1 expression in tumor cells only and were able to identify the prognostic and predictive role in TNBC patients. The association between PD-L1 expression and improved outcomes in TNBC can be partially explained by the fact that TNBC is immunologically active [46,47] or the increased chemosensitivity in immune-active TNBC [48,49].

While this study provides important information with potential impact in clinical practice about BC from Middle Eastern ethnicity, it has several limitations. First, the use of TMA, as many miss true protein expression due to intra-tumor heterogeneity, although we minimize this limitation by sampling from two representative areas in each tumor. Second, this study is retrospective, and a single-center study. Third, the majority (91%) of patients with advanced (Stage II or higher) BC were included, which might affect the influence of PD-L1 expression on prognosis. Lastly, immunohistochemical double staining with pan-cytokeratin could more accurately differentiate PD-L1 expression on tumor cells, thereby increasing the accuracy and precision of the PD-L1 measure.

## 5. Conclusions

In conclusion, our study shows that PD-L1 was an independent prognostic factor in the TNBC subgroup of BC but not in the overall cohort. Additionally, we demonstrated strong association between PD-L1 expression and mismatch repair deficiency in Middle Eastern BC patients. Overall, these findings might help in the development of more appropriate treatment strategies for BC in Middle Eastern population. Since results of PDL1 in TNBC have been contradictory, and this particular study is the first study in Middle eastern ethnicity, additional studies are needed to verify our study results.

## Figures and Tables

**Table 1:** Clinico-pathological variables for the patient cohort (n = 1009).

Clinico-Pathologic Variables	n (%)
**Age (years)**	
≤50	686 (68.0)
>50	323 (32.0)
Median (in years)	45.0
Range (IQR) ^	39.0–54.0
**Histological Type**	
Infiltrating ductal carcinoma	913 (90.5)
Infiltrating lobular carcinoma	44 (4.4)
Mucinous carcinoma	16 (1.6)
Others	36 (3.5)
**Tumor stage**	
I	91 (9.0)
II	401 (39.7)
III	379 (37.6)
IV	91 (9.0)
Unknown	47 (4.7)
**Histologic grade**	
Well-differentiated	77 (7.6)
Moderately differentiated	514 (50.9)
Poorly differentiated	405 (40.2)
Unknown	13 (1.3)
**Estrogen receptor**	
Positive	662 (65.6)
Negative	346 (34.3)
Unknown	1 (0.1)
**Progesterone receptor**	
Positive	579 (57.4)
Negative	426 (42.2)
Unknown	4 (0.4)
**Her-2 neu**	
Positive	379 (37.6)
Negative	628 (62.2)
Unknown	2 (0.2)
**Triple negative breast cancer**	
Yes	149 (14.8)
No	852 (84.4)
Unknown	8 (0.8)
**Survival duration (in months)**	
Median	48.0
Range (IQR) ^	26.0–74.0

^ IQR, inter quartile range.

**Table 2:** Correlation of PD-L1 protein expression with clinico-pathological parameters in breast cancer.

Clinico-Pathologic Variables	Total		PD-L1 Positive		PD-L1 Negative		*p* Value
	N	%	N	%	N	%	
**Total Number of Cases**	1003		329	32.8	674	67.2	
**Age Groups**							
≤50	680	67.8	237	34.9	443	65.1	0.0432 *
>50	323	32.2	92	28.5	231	71.5	
**Histology**							
Infiltrating ductal carcinoma	909	94.0	296	32.6	613	67.4	0.6176
Infiltrating lobular carcinoma	42	4.3	11	26.2	31	73.8	
Mucinous carcinoma	16	1.7	6	37.5	10	62.5	
**Histological Grade**							
Well-differentiated	76	7.7	17	22.4	59	77.6	0.0025 *
Moderately differentiated	511	51.5	151	29.6	360	70.4	
Poorly differentiated	404	40.8	155	38.4	249	61.6	
**pT**							
T1	213	22.1	68	31.9	145	68.1	0.8039
T2	484	50.2	163	33.7	321	66.3	
T3	143	14.8	42	29.4	101	70.6	
T4	124	12.9	40	32.3	84	67.7	
**pN**							
N0	307	33.2	102	33.2	205	66.8	0.2121
N1	297	32.1	102	34.3	195	65.7	
N2	192	20.8	50	26.0	142	74.0	
N3	128	13.9	44	34.4	84	65.6	
**pM**							
M0	808	89.9	265	32.8	543	67.2	0.2968
M1	91	10.1	25	27.5	66	72.5	
**Tumor Stage**							
I	91	9.5	33	36.3	58	63.7	0.6161
II	398	41.5	128	32.2	270	67.8	
III	378	39.5	126	33.3	252	66.7	
IV	91	9.5	25	27.5	66	72.5	
**Estrogen Receptor**							
Positive	656	65.5	184	28.1	472	71.9	<0.0001 *
Negative	346	34.5	145	41.9	201	58.1	
**Progesterone Receptor**							
Positive	575	57.6	161	28.0	414	72.0	0.0001 *
Negative	424	42.4	168	39.6	256	60.4	
**Her-2 neu**							
Positive	379	37.9	131	34.6	248	65.4	0.3729
Negative	622	62.1	198	31.8	424	68.2	
**Triple-Negative Breast Cancer**							
Yes	149	15.0	64	43.0	85	57.0	0.0062 *
No	846	85.0	265	31.3	581	68.7	
**Ki-67**							
High	630	64.1	238	37.8	392	62.2	<0.0001 *
Low	352	35.9	88	25.0	264	75.0	
**MMR Protein Expression**							
Deficient MMR	33	3.3	20	60.6	13	39.4	0.0009 *
Proficient MMR	970	96.7	309	31.9	661	68.1	

*, significant *p* value; MMR—mismatch repair.

**Table 3:** Correlation of PD-L1 protein expression with clinico-pathological parameters in triple-negative breast cancer.

Clinico-Pathologic Variables	Total		PD-L1 Positive		PD-L1 Negative		*p* Value
	N	%	N	%	N	%	
**Total Number of Cases**	149		67	45.0	82	55.0	
**Age Groups**							
≤50	113	75.8	52	46.0	61	54.0	0.6471
>50	36	24.2	15	41.7	21	58.3	
**Histology**							
Infiltrating Ductal Carcinoma	139	98.6	61	43.9	78	56.1	0.0711
Infiltrating Lobular Carcinoma	2	1.4	2	100.0	0	0.0	
**Histological Grade**							
Moderately differentiated	41	27.7	15	36.6	26	63.4	0.2225
Poorly differentiated	107	72.3	51	47.7	56	52.3	
**pT**							
T1	23	16.2	11	47.8	12	52.2	0.6633
T2	75	52.8	33	44.0	42	56.0	
T3	22	15.5	12	54.5	10	45.5	
T4	22	15.5	8	36.4	14	63.6	
**pN**							
N0	59	44.0	22	37.3	37	62.7	0.0459 *
N1	40	29.8	25	62.5	15	37.5	
N2	21	15.7	7	33.3	14	66.7	
N3	14	10.5	5	35.7	9	64.3	
**pM**							
M0	114	83.8	53	46.5	61	53.5	0.1987
M1	22	16.2	7	31.8	15	68.2	
**Tumor Stage**							
I	13	9.3	8	61.5	5	38.5	0.3864
II	57	40.7	26	45.6	31	54.4	
III	48	34.3	21	43.8	27	56.2	
IV	22	15.7	7	31.8	15	68.2	
**Ki-67**							
High	137	91.9	61	44.5	76	55.5	0.7153
Low	12	8.1	6	50.0	6	50.0	
**MMR Protein Expression**							
Deficient MMR	4	2.7	3	75.0	1	25.0	0.2159
Proficient MMR	145	97.3	64	44.1	81	55.9	
**Overall Survival**				83.1		62.3	0.0226 *
**Recurrence-Free Survival**				81.5		64.6	0.0169 *

*, significant *p* value; MMR—mismatch repair.

**Table 4:** Univariate and multivariate analysis of clinico-pathological variables and PD-L1 expression using the Cox proportional hazard model for overall survival and recurrence-free survival in triple-negative breast cancer.

		Overall Survival					Recurrence-Free Survival			
		Univariate		Multivariate			Univariate		Multivariate	
Clinico-Pathological Variables	Number of Events per Covariate	Hazard Ratio (95% CI)	*p*-Value	Hazard Ratio (95% CI)	*p*-Value	Number of Events per Covariate	Hazard Ratio (95% CI)	*p*-Value	Hazard Ratio (95% CI)	*p*-Value
**Age (years)** >50 ( vs. ≤ 50)	10 (vs. 27)	0.73 (0.29–1.57)	0.4519	0.27 (0.09–0.80)	0.0175 *	11 (vs. 28)	0.74 (0.30–1.60)	0.4821	0.35 (0.11–0.86)	0.0355 *
**Histology** IDC (vs. others)	26 (vs. 11)	0.48 (0.03–2.23)	0.4706	0.81 (0.10–6.67)	0.8426	28 (vs. 11)	0.26 (0.01–1.27)	0.1922	0.18 (0.01–1.03)	0.1199
**Grade** 3 (vs. 1–2)	25 (vs. 12)	0.61 (0.31–1.27)	0.1660	0.54 (0.25–1.18)	0.1241	29 (vs. 10)	1.02 (0.47–2.54)	0.9716	0.68 (0.28–1.82)	0.4119
**Lymph Node Metastasis** N1-3 (vs. N0)	25 (vs. 12)	3.34 (1.52–8.38)	0.0050 *	4.98 (1.95–12.75)	0.0008 *	24 (vs. 15)	1.82 (0.92–3.79)	0.0943	2.70 (1.31–5.87)	0.0089 *
**Stage** IV (vs. I–III)	11 (vs. 26)	3.91 (1.82–7.92)	0.0002 *	2.80 (1.21–6.45)	0.0159 *	10 (vs. 29)	0.82 (0.20–2.33)	0.7481	0.76 (0.18–2.23)	0.6593
**PD-L1** Positive (vs. Negative)	11 (vs. 26)	0.45 (0.21–0.89)	0.0272 *	0.28 (0.11–0.64)	0.0043 *	14 (vs. 25)	0.42 (0.20–0.86)	0.0205 *	0.31 (0.13–0.67)	0.0043 *

*, significant *p* value.
